# Supplementary material for: Content-rich biological network constructed by mining PubMed abstracts
Source: BMC Bioinformatics. 2004 Oct 8;5:147. doi: 10.1186/1471-2105-5-147 (PMC528731; doi:10.1186/1471-2105-5-147)
Supplement: Additional File 2 — The original results of the above study (non-essential files are deleted to keep the file size under the limit set by BMC bioinformatics). [file 1471-2105-5-147-S2.bz2 › chilibotAdditionalFile2/dip05/162ID8612709E567/html/ACVR1B_ACVR2.html]

 


 **ACVR1B** and **ACVR2** 
  
Found 46 abstracts in PubMed, retrieved 05.  
 

 What does Google say? 
 PDF only 
| .edu only 

---

**Interactive relationship** (e.g. stimulation, inhibition, etc)

**Inhibitory relationship**- A kinase deleted version of ALK4  [ **ACVR1B** ]  can form an inactive complex with activin and ActRII  [ **ACVR2** ]  IIB and thereby acts in a dominant negative manner to block activin signaling.  Ref: 12665502 J Biol Chem< MedlineTA>J Biol Chem,
**Neutral relationship**

**Non-interactive relationship** (e.g. studied together, co-existance, homology, etc.)

- Crosslinking studies measuring binding of 125I activin A to the ALK4  [ **ACVR1B** ]  trunc mutants in the presence of ActRII  [ **ACVR2** ]  implicated the same residues.  Ref: 12665502 J Biol Chem< MedlineTA>J Biol Chem,
- Surprisingly, activin had no effect on follistatin mRNA levels, despite the fact that FS D1h cells express ActRII  [ **ACVR2** ] , ActRIIB, and ALK 4  [ **ACVR1B** ]  ActRIB.  Ref: 12538636 Endocrinology, 2003
- Activins, like other members of the TGF beta superfamily, initiate signaling by assembling a complex of two types of transmembrane serine threonine receptor kinases classified as type II ActRII  [ **ACVR2** ]  or ActRIIB and type I ALK4  [ **ACVR1B** ] .  Ref: 12665502 J Biol Chem< MedlineTA>J Biol Chem, 2003
- These affinities are similar to those for binding to a chimeric receptor in which the ECD1 of CRFR2beta replaces the ECD of the type 1B activin receptor  [ **ACVR2** ]  ALK4  [ **ACVR1B** ] .  Ref: 12611895 J Biol Chem, 2003
